# Case Report: Paradoxical responses to pasireotide in a patient with a silent corticotroph adenoma that transformed into an ACTH-secreting adenoma

**DOI:** 10.3389/fonc.2026.1712093

**Published:** 2026-02-18

**Authors:** Anna Brona, Aleksandra Zdrojowy-Wełna, Marek Bolanowski

**Affiliations:** Department and Clinic of Endocrinology and Internal Medicine, Wroclaw Medical University, Wroclaw, Poland

**Keywords:** case report, Cushing’s disease, paradoxical response, pasireotide, silent corticotroph adenoma

## Abstract

**Introduction:**

There are only a few reported cases of paradoxical response to pasireotide treatment of adrenocorticotropic hormone (ACTH)-secreting tumors, mostly aggressive macroadenomas, which led to therapy discontinuation. We present a case of a patient with Cushing’s disease and paradoxical responses to pasireotide Long-Acting Release (LAR) in whom, despite the initial increase in hypercortisolemia, the treatment was successful in terms of tumor shrinkage. The rarity of paradoxical responses to pasireotide LAR makes this case a valuable insight into a potentially dangerous and poorly understood clinical situation.

**Case description:**

The patient was diagnosed in 2008 with a non-secreting pituitary macroadenoma, causing only neurological symptoms. Initial therapy included two transsphenoidal surgeries and stereotactic radiotherapy. The pathological report indicated ACTH expression, although it was clinically silent. After 7 years, the patient underwent reoperation due to sudden deterioration of the neurological condition and regrowth of the tumor. In 2019, hypercortisolemia appeared, and treatment with pasireotide LAR was initiated. Pharmacotherapy continued for 4 years; four of eight pasireotide LAR injections were followed by paradoxical responses, which were managed with steroidogenesis inhibitors. During this therapy, the tumor size was significantly reduced.

**Conclusions:**

Pasireotide LAR successfully reduced the tumor mass despite paradoxical responses. A transient increase in hypercortisolemia after pasireotide LAR was difficult to predict but was managed with steroidogenesis inhibitors.

## Introduction

Adrenocorticotropic hormone (ACTH)-secreting adenoma [Cushing’s disease (CD)] is an endocrine disorder caused by excessive production of ACTH by a pituitary tumor, resulting in hypercortisolemia. CD leads to numerous metabolic and cardiovascular complications, including hypertension, diabetes, coronary heart disease, as well as osteoporosis, infections, and thromboembolism, collectively increasing mortality in affected patients (1). The primary treatment for ACTH-secreting pituitary tumors is surgery. Pasireotide, a multireceptor somatostatin ligand that primarily targets somatostatin receptor 5 (SSTR 5), is used for pharmacological treatment directed at the tumor (2, 3).

Silent corticotroph adenomas are a rare subtype of corticotroph adenomas with a high risk of recurrence (4, 5). They are commonly referred to as aggressive pituitary tumors (APT) because of radiological invasiveness, a rapid tumor growth rate, or clinically relevant tumor growth despite optimal standard treatments (6). Surgery is typically performed, followed by radiotherapy and temozolomide (4). Only one study on silent corticotroph adenomas has investigated the response to pasireotide; no paradoxical response or tumor shrinkage was reported in this group (7). There are a few descriptions of paradoxical responses to pasireotide in the literature (8, 9). We present a case report of paradoxical responses to pasireotide LAR in a patient with a silent corticotroph adenoma that transformed into an ACTH-secreting adenoma.

## Case description

We present a case of a 69-year-old man with Cushing’s disease occurring 11 years after being diagnosed with an aggressive silent corticotroph adenoma (**Table 1**).

**Table 1:** Timetable of the treatment.

2008	Neurological symptoms, first surgery, diagnosis of silent corticotroph adenoma, pituitary insufficiency
2009	Second surgery, radiotherapy
2017	Third surgery
June 2019	Onset of clinical hypercortisolemia
September 2019	First dose of pasireotide LAR, first paradoxical response
October 2019	Second dose of pasireotide LAR
November 2019	Third dose of pasireotide LAR
March 2020	Fourth dose of pasireotide LAR
June 2020	Fifth dose of pasireotide LAR
March 2021	Sixth dose of pasireotide LAR, second paradoxical response
April 2021	Seventh dose of pasireotide LAR, third paradoxical response
October 2022	Eighth dose of pasireotide LAR, fourth paradoxical response

The patient was first diagnosed with a non-secreting pituitary macroadenoma (25 mm × 16 mm × 15 mm) in February 2008 at the age of 55 (**Table 2**). He complained of diplopia and eyelid drooping caused by paresis of oculomotor nerves III and VI, which progressed particularly on the right side. The tumor caused destruction of the sella turcica, demonstrated invasive growth into the sphenoid sinus, and extended into the cavernous sinus. At that time, the patient did not exhibit symptoms of hypercortisolemia. ACTH concentration and the diurnal rhythm of cortisol were within normal limits. Family history was noncontributory.

The patient underwent transsphenoidal surgery in March 2008 and was reoperated on in 2009.

After surgery, the mobility of the right eye improved, and the eyelid was elevated.

Pathology reported a densely granulated corticotroph adenoma, and clinical data suggested a silent corticotroph adenoma with Crooke cells. The Molecular Immunology Bortel Index (MIB-1) was approximately 3%. The percentage of Crook cells was not specified. The patient underwent stereotactic radiotherapy in the same year due to tumor recurrence (photons X 6 MV for the pituitary gland, with a total dose of 50 Gray). After the surgeries, pituitary insufficiency (panhypopituitarism) was diagnosed, and the patient started supplementation with l-thyroxine and hydrocortisone.

The recurrence of the tumor was noticed on magnetic resonance imaging (MRI) in March 2016. The tumor measured 15 mm × 17 mm × 19 mm and was located in the sella turcica, invading the suprasellar cistern, compressing the infundibulum, and the optic chiasm. It encapsulated one-third of the circumference of the cavernous sinus (**Table 2**). In April 2017, the patient was admitted to the neurosurgery department due to symptoms of intratumoral hemorrhage, including sudden pain in the right eye, eyelid drooping, and ocular nerve palsy. An urgent craniotomy was subsequently performed.

**Figure 1:**
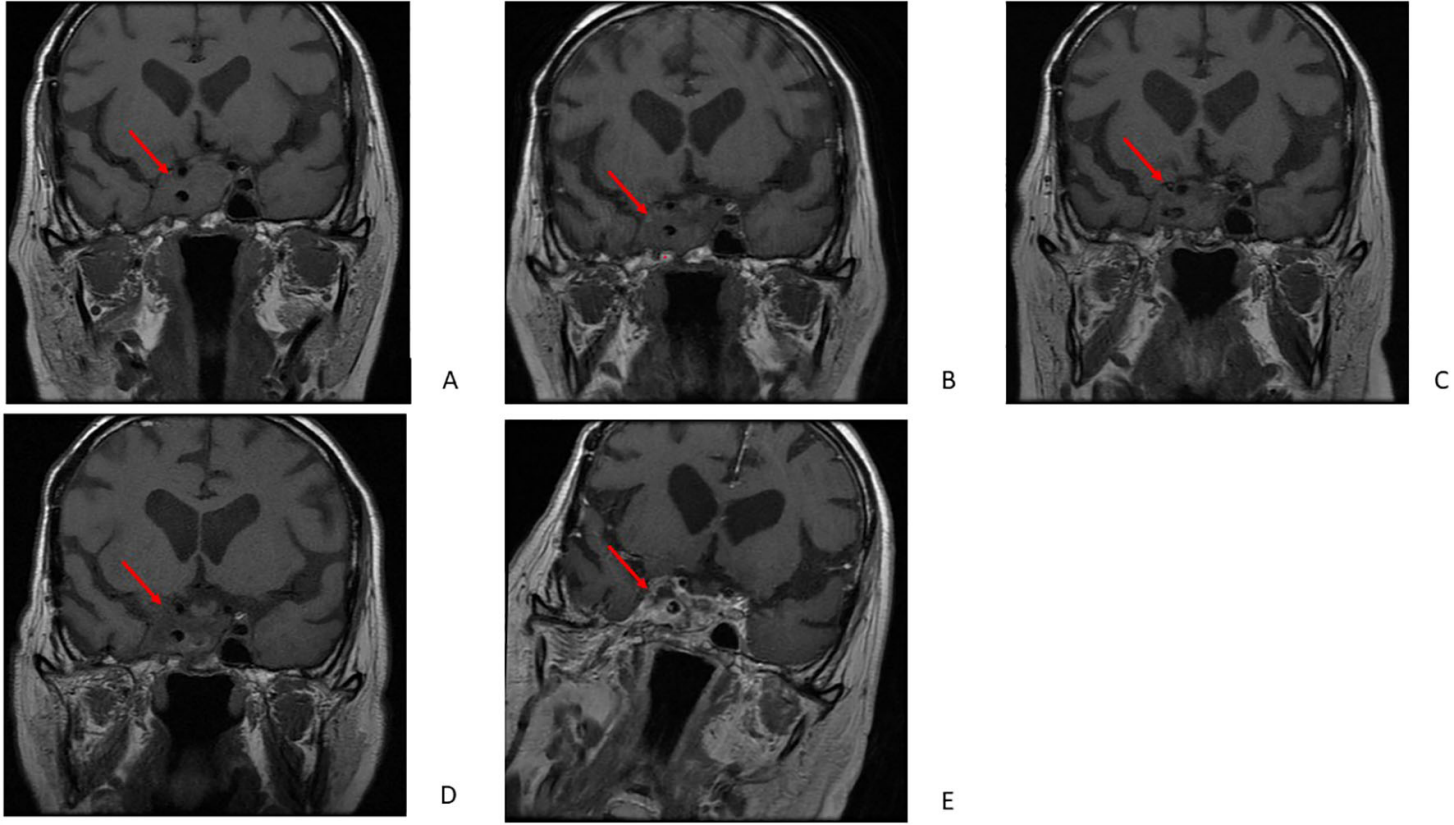
MRI examinations. The images show the patient’s coronal MRI of the pituitary tumor in July 2018 **(A)**, December 2019 **(B)**, February 2021 **(C)**, March 2022 **(D)**, and August 2022 **(E)**.

**Figure 2:**
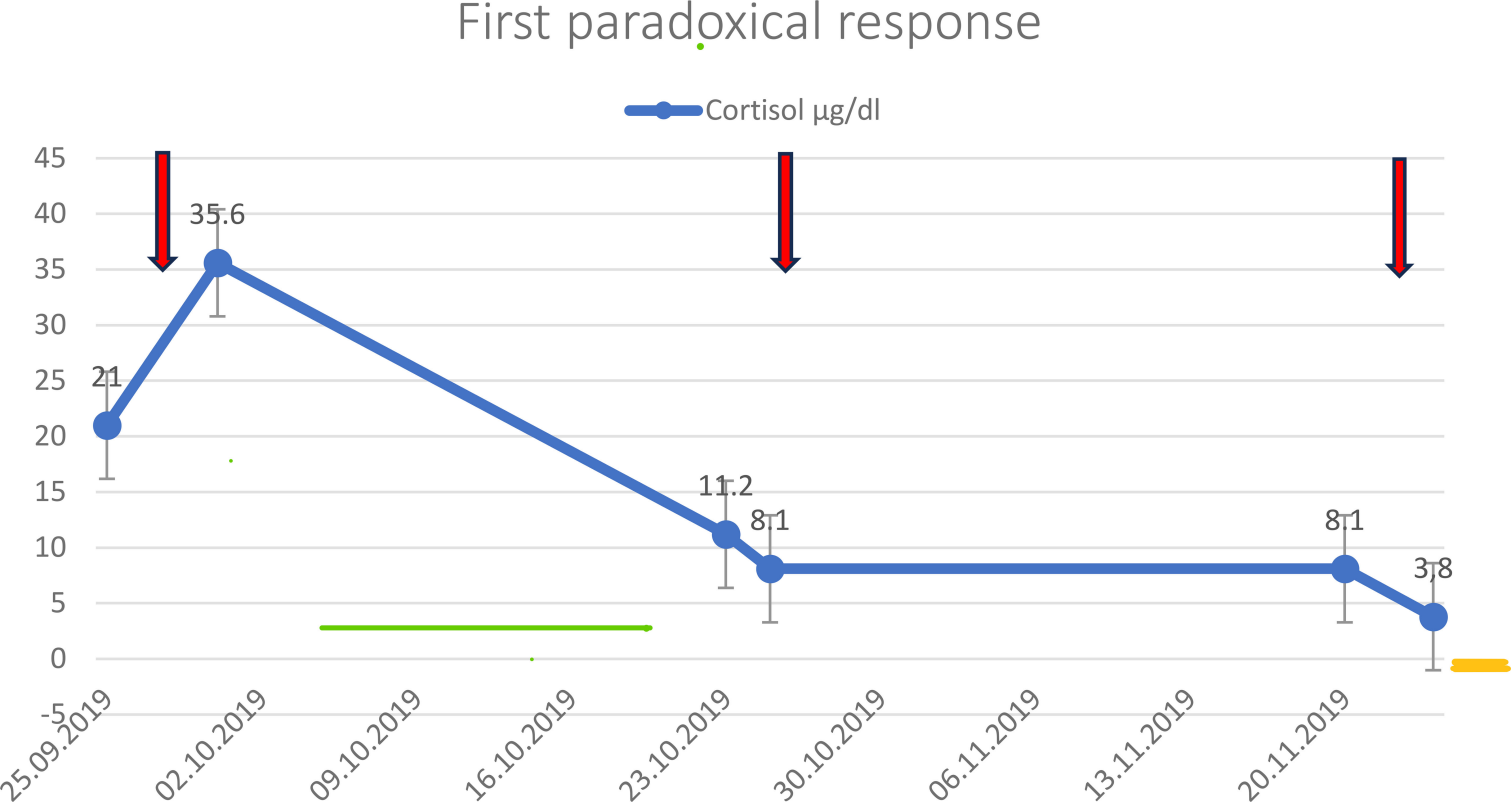
First paradoxical response: cortisol level (µg/dl), administration of pasireotide LAR (arrows), metyrapone (green line), and hydrocortisone (yellow line) in 2019.

Despite the third surgery, the tumor continued to grow, infiltrating the skull base and adhering to the optic chiasm and optic canal in July 2018. The tumor measured 32 mm × 22 mm × 31 mm; it enlarged further, destroying the floor of the sella turcica and extending into the sphenoid sinus, clivus, and the right Meckel’s cave (**Figure 1A**, **Table 2**). Surgery was not feasible due to the tumor size and location. Rapid deterioration ruled out radiotherapy. After a few months, hydrocortisone treatment was discontinued, and mild hypercortisolism was first detected in laboratory results, although clinical signs were absent. In 2019, CD was confirmed by the dexamethasone suppression test, and pasireotide LAR at a dose of 20 mg was administered as emergency treatment. In September 2019, serum cortisol level was 21 µg/dl (3.7–19.4), and ACTH was 107 pg/ml (0–46) before administration of pasireotide LAR, increasing to 35.6 µg/dl and 176 pg/ml after injection, respectively, representing the first paradoxical reaction to pasireotide LAR (**Figure 1**, **Table 1**). Metyrapone treatment was started at a dose of 500 mg/day and was withdrawn after 2 weeks due to cortisol normalization. Subsequent cortisol and ACTH measurements were obtained in October and November 2019, before and after administration of the second and third doses of 20 mg pasireotide LAR. These measurements did not reveal either recurrence of hypercortisolemia or paradoxical response to pasireotide LAR (**Figure 2**). After the third dose, 10 mg of hydrocortisone was introduced for a month due to endogenous morning cortisol levels below 10 µg/dl. MRI of the pituitary was performed in December 2019 showed marked regression of the pituitary tumor in the sphenoid sinus, nasal cavity, and right cavernous sinus. Contrast enhancement decreased compared with the previous MRI, suggesting progression of degeneration (**Figure 1B**, **Table 2**).

**Table 2:** Descriptions of MRI examinations from 2008 to 2022.

MRI 2008	Intrasellar part 25 mm × 16 mm × 15 mm	Invasive growth into the sphenoid sinus; access to the cavernous sinus	
MRI 2016	Intrasellar part 15 mm × 17 mm × 19 mm	Encapsulation of one-third of the circumference of the cavernous sinus; invasion of the suprasellar cistern	Compression of the optic chiasm
MRI 2018	Intrasellar part 32 mm × 22 mm × 31 mm	Invasive growth and extension into the cavernous sinuses; access to the sphenoid sinus, clivus, and Meckel’s cave	Adherence to the optic chiasm; growth around the right optic canal
MRI 2019	Intrasellar part unchanged; degeneration of adenoma	Marked regression of the tumor within the sphenoid sinus (14 mm × 11 mm × 10 mm) and the right cavernous sinus	Marked regression of tumor mass in the superior and medial nasal duct; invasion of the right optic nerve canal
MRI 2021	Cystic degeneration in the intrasellar tumor mass; slight decrease in the intrasellar part	Progression in the superior part of the sinus cavernous, 16 mm × 10 mm × 10 mm; growth toward the right trigeminal nerve and to the pontine cistern 10 mm × 10 mm × 11 mm, slightly decreased in the right sphenoid sinus (12 mm × 11 mm × 10 mm)	Compression of the medial part of the temporal lobe; adherence to the right middle cerebral artery; invasion of the right optic canal
MRI February 2022	Heterogeneous mass, with cystic degeneration; further regression of intrasellar mass	Invasion of the right sinus cavernous, enclosure of the cavernous part of the right internal carotid artery; complete regression of solid tumor mass beyond the superior part of the right cavernous sinus and within the superior part of the right cavernous sinus; partial regression of tumor mass extending posteriorly to the right cavernous sinus; invasion in the right sphenoid sinus; invasion of trigeminal nerve; invasion of pontine cistern (6 mm × 4 mm × 8 mm)	Adherence to the right optic canal (decreased mass)

The fourth and fifth doses of 20 mg pasireotide LAR in March and June 2020 did not require additional management. During this period, follow-up was reduced mainly to clinical assessment due to the coronavirus disease 2019 (COVID-19) pandemic. Cortisol levels were measured only before the administration of pasireotide LAR (7.7 µg/dl in March 2020 and 8.4 µg/dl in June 2020).

**Figure 3:**
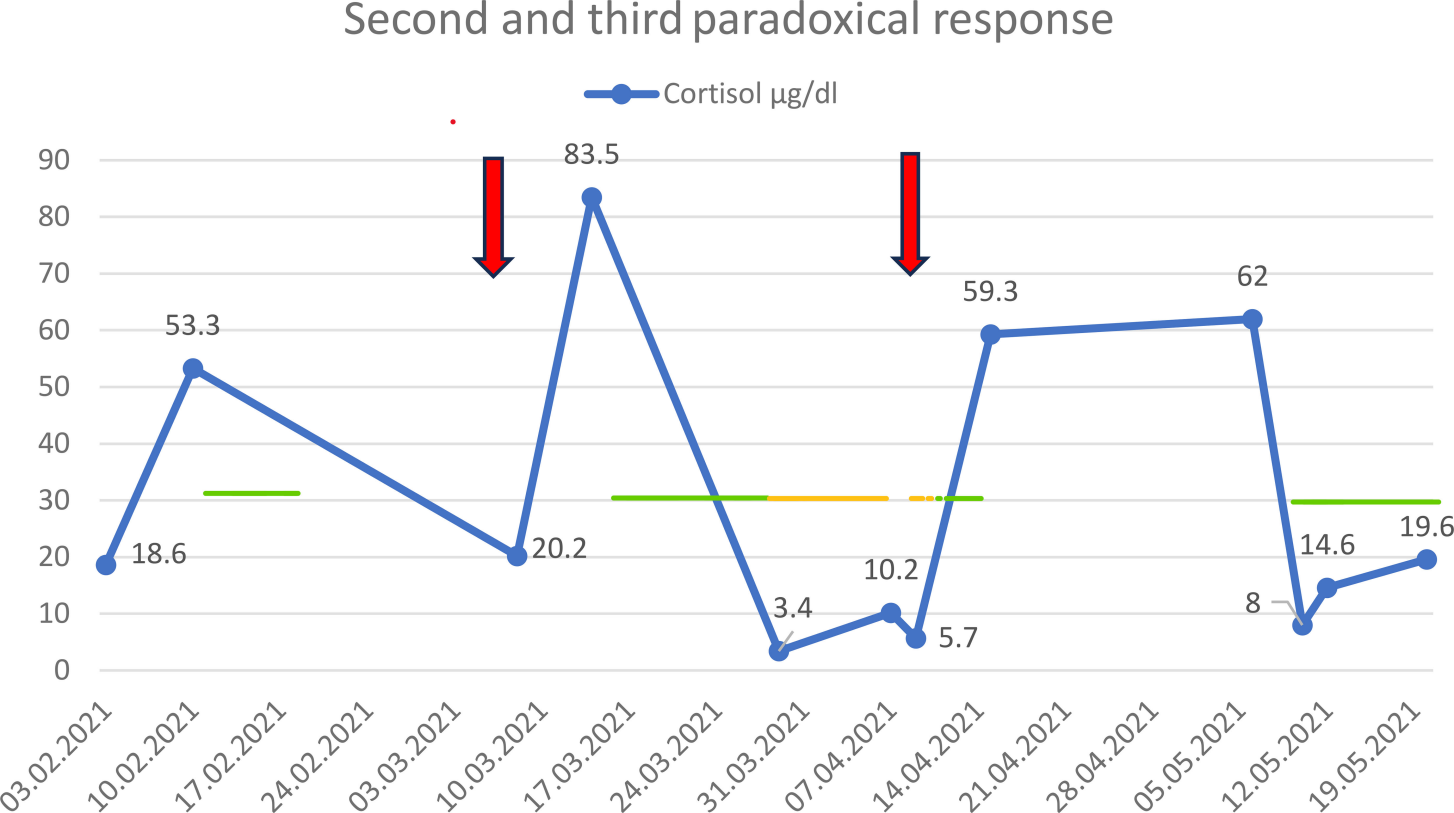
Second and third paradoxical responses: cortisol level and administration of pasireotide LAR (arrows), metyrapone (green line), and hydrocortisone (yellow line) in 2021.

In February 2021, hypercortisolemia recurred (serum cortisol up to 53.3 µg/dl, Urinary Free Cortisol (UFC) up to 2,170.8 µg/24 h). This was followed by progression of a pituitary adenoma in the superior part of the right cavernous sinus, with compression of the medial part of the temporal lobe. The tumor mass in the right sphenoid sinus slightly decreased. After contrast administration, the adenoma enhanced in a heterogeneous, mainly peripheral pattern, suggesting cystic degeneration in the intrasellar tumor mass. A tumor mass was also present in the right optic canal (**Figure 1**, **Table 2**). In March 2021, the sixth dose of 20 mg pasireotide LAR was administered. Within 3 days after administration, cortisol level was 20.2 µg/dl, ACTH 223 pg/ml, and 24-UFC 437.8 µg/24 h. About a week later, all parameters increased (cortisol up to 83.5 µg/dl, ACTH 392 pg/ml, 24-UFC 7391 µg/24 h), indicating a second paradoxical reaction (**Figure 3**). Therapy with metyrapone was started at an initial dose of 250 mg and titrated; the maximal dose was 1,000 mg/day before being reduced. The total therapy duration was 19 days. Subsequently, due to low cortisol level (3.4 μg/dl), replacement therapy with 10 mg of hydrocortisone was administered.

In April 2021, the seventh dose of 20 mg pasireotide LAR was administered. Cortisol levels initially were 10.2 µg/dl and increased to 59.3 µg/dl after a week, while ACTH remained more stable (149 and 186 pg/ml, respectively), and the third paradoxical response was diagnosed. The patient received metyrapone in small doses (250–500 mg) for a few days due to a shortage of the drug. He was also instructed to withdraw hydrocortisone (**Figure 3**). Cortisol values normalized after a month, when metyrapone was again administered in May 2021 (500 mg for 4 days and 250 mg for a week). Subsequently, the patient did not require any steroidogenesis inhibitor or hydrocortisone until August 2022.

In March 2022, MRI revealed complete regression of the solid tumor mass beyond and within the superior part of the right cavernous sinus. Partial regression of the tumor mass extending posteriorly to the right cavernous sinus was observed. Furthermore, regression of the intrasellar tumor, the tumor invading the right cavernous sinus, the tumor enclosing the cavernous part of the right internal carotid artery (ICA), and the tumor in the right sphenoid sinus was noted. The tumor mass was heterogeneous, with cystic degeneration and mainly peripheral heterogeneous enhancement. The remnant tumor adhered to the right optic canal (**Figure 1D**, **Table 2**). Substantial regression of the tumor mass with degeneration was diagnosed.

**Figure 4:**
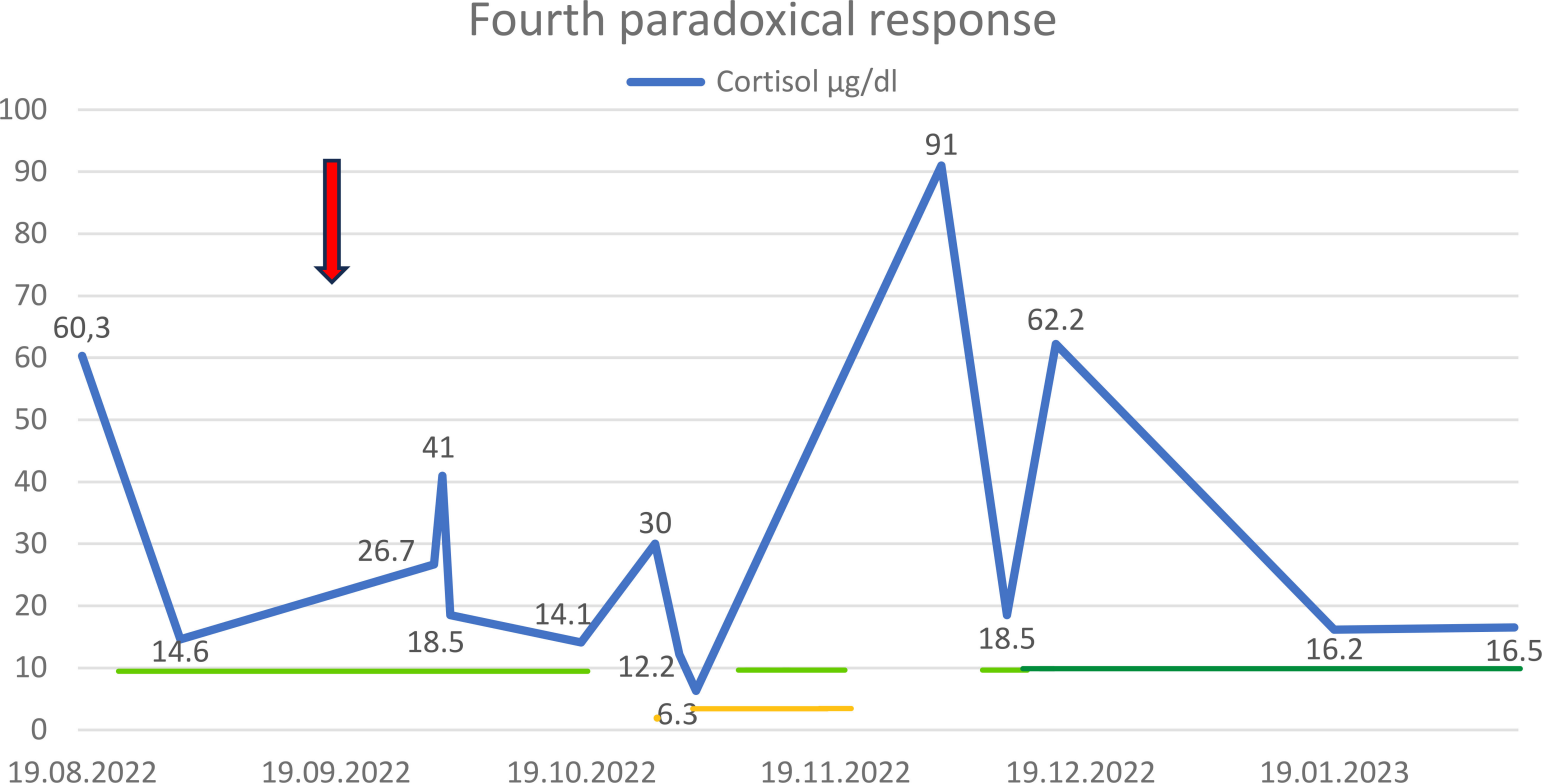
Fourth paradoxical response: cortisol level and administration of pasireotide LAR (arrow), metyrapone (green line), osilodrostat (dark green line), and hydrocortisone (yellow line) in 2022 and 2023.

In August 2022, there was a recurrence of severe hypercortisolemia, which was treated with metyrapone (initially 750 mg/day, later 500 mg/day) (**Figure 4**). MRI revealed no significant change in tumor mass (**Figure 1E**, **Table 2**). In October 2022, the patient received the eighth dose of 20 mg pasireotide LAR while on metyrapone. Three days after the injection, serum cortisol levels remained within the upper reference range, while UFC increased (from 489.6 to 1,785 µg/24 h). The fourth paradoxical response was diagnosed, and the dose of metyrapone was increased to 1,500 mg/day. About 2 weeks later, the dose was reduced, and 2 days afterward it was withdrawn. The following day, metyrapone was immediately readministered (750 mg) due to recurrence of hypercortisolemia, and after 3 days, the dose was increased. Finally, after 31 days of hospitalization and metyrapone therapy, the patient was discharged on metyrapone 1,000 mg/day. Dose adjustment was limited by the increasing activity of gamma-glutamyl-transpeptidase (GGTP). Simultaneously, therapy with 30 mg of hydrocortisone was started. The patient continued treatment for the next 20 days.

In December 2022, he was admitted again to the endocrinology department with very high concentrations of cortisol (serum cortisol 91 µg/dl, 24-UFC 4,140 µg/24 h, and ACTH 816 pg/ml). The patient received up to 750 mg of metyrapone/day for 4 days. Additionally, therapy with osilodrostat was initiated at 2 mg/day, with the highest dose reaching 7 mg/day. Osilodrostat was administered for 2 months and 1 week, with the last dose being 5 mg/day. In January 2023, the patient attended the final follow-up, and the serum cortisol level was normal (10.6 µg/dl). In February 2023, the patient died of cardiovascular complications. A consolidated timeline table summarizing the onset of symptoms, surgeries, radiotherapy, pasireotide LAR injections, and paradoxical responses is presented in **Table 1**.

In summary, the patient received eight doses of 20 mg pasireotide LAR between September 2019 and October 2022. There were four paradoxical responses: the first occurred in September 2019 (**Figure 2**), the second and third between March and May 2021 (**Figure 3**), and the fourth in October 2022 (**Figure 4**). Each paradoxical reaction was followed by transient clinical symptoms, including hypokalemia, hyperglycemia, and lower leg edema. The incidence of the first paradoxical response was difficult to predict. Subsequent responses were anticipated, but no data suggested any preventive actions. Each paradoxical response to pasireotide LAR was managed with metyrapone. The dose was adjusted according to cortisol concentrations in both serum and 24-UFC. Due to multimorbidity and liver involvement, the dose of metyrapone was kept as low as possible. Episodes of limited access to the medication occurred because of the high cost for outpatient clinic patients. Osilodrostat had not been available before the end of 2022 due to administrative issues. Since the therapy with osilodrostat began, the patient could receive it at both home and the hospital. It was also expected to be better tolerated, particularly regarding transaminase activity.

During 14 years of treatment, the patient was diagnosed with multiple concomitant diseases: hypopituitarism, atrial fibrillation, implantation of a dual-chamber DDD pacemaker due to tachy-brady syndrome, coronary heart disease, heart failure, diabetes, osteoporosis, ulcerations of the distal esophagus, Barrett’s esophagus, gastritis and duodenitis, and iron deficiency anemia. In addition to the multimorbidity, another challenge was limited access to MRI due to the pacemaker. Each MRI required special arrangements with the cardiology and radiology teams.

## Discussion

This case report presents different patterns of paradoxical responses to pasireotide LAR in a single patient. It also demonstrates effective treatment of an aggressive pituitary tumor in terms of tumor shrinkage. The report describes a very rare clinical situation of hypercortisolemia in a patient with a silent corticotroph adenoma approximately 10 years after the initial diagnosis of a nonfunctioning pituitary adenoma.

Since there are no data on how to manage paradoxical reactions to pasireotide, therapy was personalized, also considering the patient’s multimorbidity and the aggressiveness of the tumor. A limitation of this case was the inability to administer temozolomide, which was unavailable at that time in our department, and shows a response rate of about 56% in aggressive corticotroph tumors. Therefore, pasireotide LAR was used as tumor-targeted therapy, also with the potential to normalize cortisol levels in 41% of ACTH-secreting tumors (1). This treatment was complicated by a paradoxical reaction to pasireotide LAR, which was successfully managed with steroidogenesis inhibitors, and pasireotide LAR was continued due to its positive effect on tumor shrinkage.

A paradoxical drug reaction produces a result opposite to the expected outcome (10). To the best of our knowledge, only two studies describe four patients with paradoxical and atypical responses to pasireotide in Cushing’s disease (8, 9).

The first case is a 57-year-old man with an ACTH-secreting pituitary tumor. Like our patient, he underwent two surgeries and radiotherapy, which contributed to the normalization of his cortisol level for a few years. When inhibitors of steroidogenesis could not be continued due to a significant increase in liver enzymes and tumor enlargement, pasireotide was introduced. A paradoxical response with an enormous increase in UFC occurred, and cortisol levels increased with escalating doses. Hypercortisolemia resolved after metyrapone. This paradoxical response and its resolution are similar to our patient’s first paradoxical response in terms of duration and management. However, urinary free cortisol was much higher than in our patient, and short-acting pasireotide was used. The main difference is that this patient underwent reoperation, and stereotactic surgery was applied; continuation of pasireotide was unnecessary. Tumor biology may also differ, as the authors observed clinical features of pituitary carcinoma. In the Greenman et al. case series, two other patients, after an initial good response to pasireotide, presented with increasing ACTH level and hypercortisolemia, respectively (8). Short-acting pasireotide was administered. Both patients were diagnosed with invasive corticotropinomas, and the first patient responded well for 2 months. In the second case, pasireotide was effective for only 1 month. Both patients required additional treatment to control hypercortisolemia and reduce tumor size. However, these were escapes rather than paradoxical responses, as treatment initiation was associated with decreased cortisol levels.

In another case report by Tamaki et al., an invasive ACTH-secreting tumor was treated with pasireotide LAR preoperatively. Short-lasting increases in ACTH and cortisol were reported after the third dose. No inhibitor of steroidogenesis was applied, and hypersecretion resolved spontaneously (9). Shortly before hypercortisolemia, the authors observed high signal activity in part of the tumor on MRI. Subsequently, tumor shrinkage was noted. In our patient, the MRI scheme was different. However, 8 months after the fifth dose of pasireotide LAR and 10 months after the seventh dose, marked cystic degeneration of the intrasellar tumor mass was observed. MRI performed after the fifth dose also showed progression in other parts of the tumor, while MRI after the seventh dose showed regression in most of those parts (**Table 2**). The seventh dose was followed by a third paradoxical response, which is supposed to resolve spontaneously, similar to the case reported by Tamaki et al.

Spontaneous resolution of hypercortisolemia is observed in cyclic ACTH-secreting pituitary tumors, which was not applicable in this case. This phenomenon may be explained by an intense response to pasireotide LAR, which caused marked destruction in tumor cells, resulting in excessive ACTH release (paradoxical response). Degeneration of the tumor mass was subsequently observed on MRI scans. During follow-up, inhibition prevailed over stimulation, leading to decreased cortisol levels.

The mechanism of paradoxical reaction to pasireotide is unknown. ACTH and cortisol elevation in this patient suggested a central (at the pituitary) level of paradoxical response. It has been shown that drugs may produce different effects due to various receptor subpopulations, receptor desensitization, multiple receptor states, and induction of competing downstream pathways of a given receptor. In general, paradoxical reactions result from multiple mechanisms (10).

Pasireotide binds to somatostatin receptors (SSTRs) on the pituitary gland and inhibits ACTH secretion. It has a higher affinity for SSTR5, which makes it more effective in some cases of Cushing’s disease (11).

Studies performed *in vitro* have shown that multireceptor ligands may activate or antagonize distinct signaling pathways, potentially altering the clinical response (8).

The various effects of multireceptor ligands, including pasireotide, on different cell lines were examined in the study by Cescato et al. (12). Pasireotide, as a somatostatin analog, is expected to inhibit cAMP production, stimulate intracellular calcium accumulation, and increase ERK phosphorylation. This study demonstrated that pasireotide does not promote intracellular calcium accumulation and acts as a partial agonist/antagonist for ERK phosphorylation. These findings support the hypothesis that the same molecule may exert antagonistic effects and contribute to different biological responses in a single patient, such as enhanced ACTH secretion followed by decreased production (12).

Additionally, endogenous somatostatin receptors are associated with more than one subtype of G proteins, which promote unique signaling pathways (13, 14).

Although pasireotide does not bind to Corticotropin-Releasing Hormone (CRH) receptors or vasopressin (AVP) receptors, which can also be present in ACTH-secreting tumors, it may make their actions more visible (at least temporarily) through inhibitory effects dependent on somatostatin receptors (12).

Interestingly, pasireotide significantly increased baseline cortisol secretion in primary cultures of normal human adrenal glands but did not affect ACTH-stimulated secretion (15).

It is likely that genetic mutations or epigenetic changes in the tumor cells may alter the response to pasireotide. The latter could be particularly important for silent corticotroph adenomas, which may transform into hypercortisolemia (8).

We believe that SSTR heterogeneity and the promotion of CRH or AVP secretion, followed by a transient overproduction of ACTH, were the most likely explanation for the paradoxical response in our case.

It has also been suggested that a transient increase in ACTH and cortisol may result from necrosis caused by pasireotide (9).

Paradoxical responses may be associated with changes in tumor structure after radiotherapy (both our patient and the patient in the Greenman et al. study underwent radiotherapy). Radiotherapy is applied to promote shrinkage and fibrosis of the pituitary tumor, resulting in changes to the cellular structure. Regrowth of the tumor likely originates from these modified cells. There are no data on how radiotherapy might influence the response to pasireotide treatment.

In our case, management of paradoxical responses was challenging. It was impossible to predict if they would appear or how long they would last, as each course was different. The most noticeable adverse event was the elevation of transaminase activity, particularly during the fourth paradoxical response, which was the longest. Finally, all episodes were addressed and followed by decreased cortisol concentrations. On three occasions, it was possible to discontinue treatment with metyrapone. The course of the last episode suggested that administration of osilodrostat could have been ceased sooner. Nevertheless, due to the presence of an aggressive pituitary tumor, complications of hypercortisolemia, and multimorbidity, long-lasting improvement could not be achieved. Despite managing paradoxical responses, the main goal of treatment was to prevent tumor growth. Pasireotide LAR was efficient in reducing tumor size between September 2019 and August 2022. In September 2022, pituitary MRI suggested tumor progression. The same observation was noted after the last MRI in January 2023. However, the progression was markedly smaller than the regrowth observed previously. We believe that the effects of treatment over the last 4 years (i.e., smaller tumor progression) are likely attributable mostly to pasireotide LAR, as earlier (between 2016 and 2019), marked tumor growth occurred despite radiotherapy.

Due to the potential risk associated with paradoxical responses, alternative treatment options were considered in our case, particularly after the third and fourth episodes. Surgery was dismissed because of expected difficulties, as it would have been the fourth procedure, and the patient’s condition was poor at that time. The effect of radiotherapy would have been observed over years rather than weeks or months. Steroidogenesis inhibitors would not have stopped tumor growth.

Paradoxical responses were followed by tumor regression, which was the most important criterion for continuing therapy despite these events.

Liver function was another important limitation to treatment in this case, beginning in October 2022. The most affected parameter was GGTP, which reached eight times the upper reference range during combined metyrapone and osilodrostat therapy. Doses had to be adjusted during the management of paradoxical reactions.

Another interesting aspect of our case was the transformation of a previously silent corticotroph adenoma into an ACTH-secreting tumor. This transformation may be related to increased expression of PCSK1 and PC1/3. Additionally, despite low PC1/3 activity in silent corticotroph adenomas, it may produce ACTH from proopiomelanocortin (POMC) over time. A third possible mechanism is the activation of POMC through sequential hypomethylation of the second promoter, leading to enhanced ACTH secretion (16).

Our case description has some important limitations. This is a single case report; our findings, particularly the repeated paradoxical responses to pasireotide LAR, cannot be generalized. Causality between pasireotide LAR and hormonal fluctuations cannot be definitively established, and tumor dedifferentiation is also possible. Due to disease severity and rarity, together with the patient’s multimorbidity, he received complex treatment for hypercortisolemia (steroidogenesis inhibitors, radiotherapy, and hydrocortisone replacement therapy), which makes it difficult to clearly attribute cortisol fluctuations to a single intervention. The timing of the cortisol increases directly after pasireotide LAR injections suggests that the paradoxical response is the most likely explanation. The standards and possibilities of pathological reporting were different in 2008, so we do not have information about important prognostic markers such as the exact Crooke cell percentage, p53 status, detailed Ki-67 distribution, or transcription factors such as T-PIT in the tumor, which would have been very helpful. Molecular or receptor-level analyses (e.g., SSTR expression profiling) might have been helpful to explain the repeated paradoxical ACTH/cortisol increases. The patient also lacked optimal compliance, and follow-up visits were irregular, particularly during the COVID-19 pandemic. The patient’s death from cardiovascular complications limits the assessment of long-term endocrine control and the survival impact of the applied therapies. Finally, other therapeutic interventions, particularly temozolomide administration, might have been beneficial in this case, but were not possible due to limited availability and the patient’s general condition at that time.

In conclusion, pasireotide LAR may be an effective treatment for a silent corticotroph adenoma that transformed into an ACTH-secreting adenoma, particularly with regard to tumor size.

Our case demonstrates that pasireotide LAR treatment may be continued despite a paradoxical reaction but requires close monitoring and frequent follow-up. Management of hypercortisolemia was challenging, yet it was resolved effectively. There is insufficient evidence for the preemptive use of steroidogenesis inhibitors prior to administration of pasireotide LAR; therefore, cortisol levels should be monitored after this treatment in patients with ACTH-secreting adenomas.

## Data Availability

The raw data supporting the conclusions of this article will be made available by the authors, without undue reservation.
